# Supplementary material for: HIV-1 Intasomes Assembled with Excess Integrase C-Terminal Domain Protein Facilitate Structural Studies by Cryo-EM and Reveal the Role of the Integrase C-Terminal Tail in HIV-1 Integration
Source: Viruses. 2024 Jul 20;16(7):1166. doi: 10.3390/v16071166 (PMC11281638; doi:10.3390/v16071166)
Supplement: Supplementary file 1 [file viruses-16-01166-s001.zip › viruses-3101545-supplementary.pdf]

Supplementary Material

Table S1. Statistics of cryo-EM data collection and structure refinement of hetero-intasomes

**Data collection and processing**	**HIV-1 CIC**
EMDB code	EMD-45364
Pixel size (Å)	0.83
Defocus range (μm)	-0.6 to -2.4
Voltage (kV)/Camera	300/K3
Total electron dose (e^-/ $Å^2$)	50
Symmetry imposed	C2
Extracted particles (no.)	940,736
Particles used final reconstruction (no.)	428,779
Final map resolution (Å, FSC=0.143)	2.01
Map sharpening factor ($Å^2$)	-40.98
**Model refinement and validation**	
PDB code	9C9M
CC_mask	0.81
CC_volume	0.80
CC_peaks	0.73
Average B-factor ($Å^2$) (Protein/Nucleotide/Ligand)	42.06/45.21/7.94
RMSD Bond lengths (Å)	0.003
RMSD Bond angles (°)	0.531
Ramachandran Most favored (%)	98.18
Allowed (%)	1.82
Disallowed (%)	0.00
Clashscore	4.08
Molprobity score	1.19
Rotamer outliers (%)	0.19
C-beta outliers	0.00

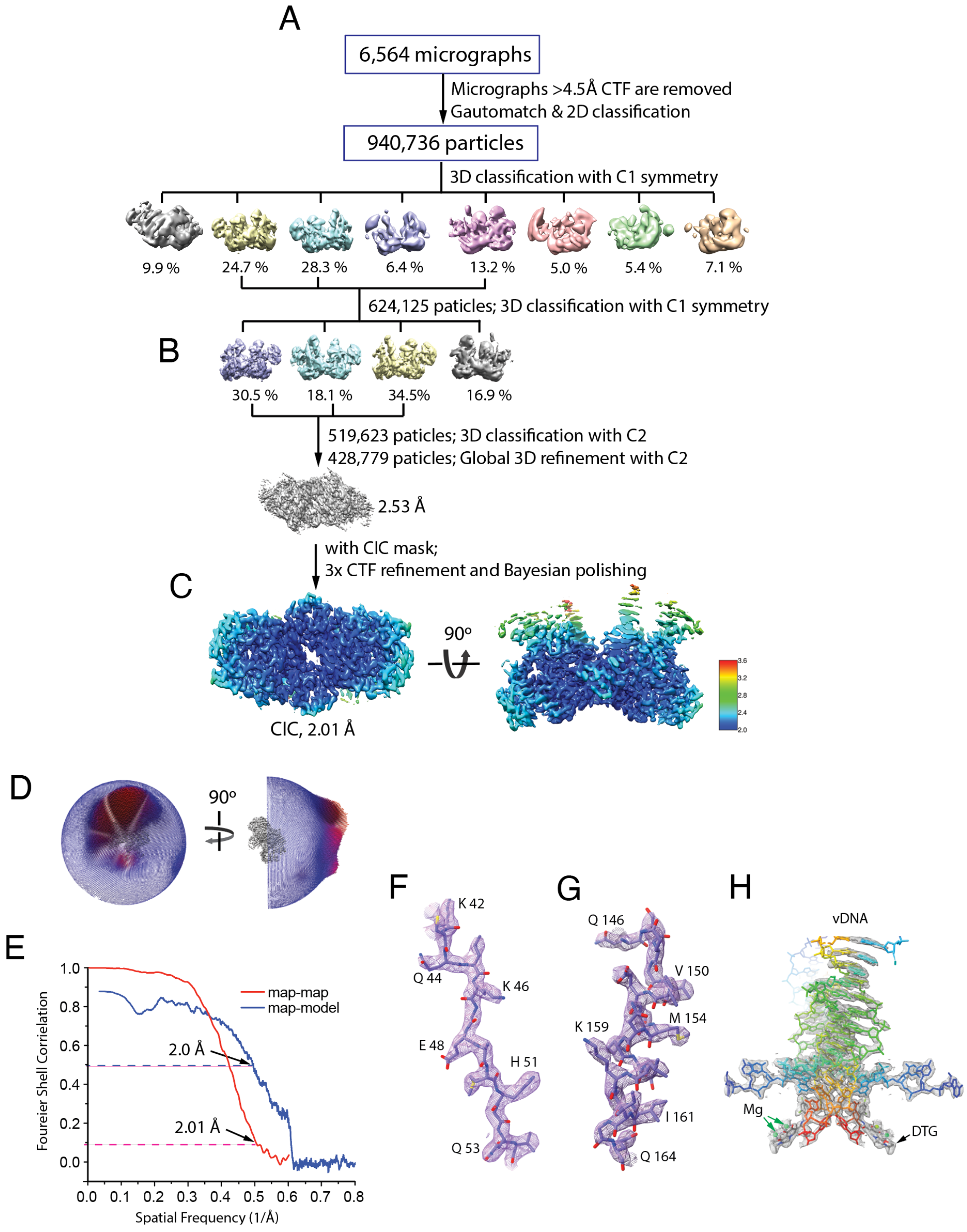

Figure S1. Structure determination of "hetero-intasomes". (**A**) Flow chart of data processing of the cryo-EM dataset. (**B**) 3D classification showed intasome assembly was very heterogeneous. Global 3D refinement of the selected particles resulted in a map at 2.53 Å with C2 symmetry imposed. Focused refinement with CTF refinement and Bayesian polishing yielded a final map at 2.01 Å. (**C**) Surface presentation of the CIC map colored according to the local resolution estimated by ResMap with the scale bar on the side. (**D**) Angular distribution of final reconstruction. (**E**) Gold-standard Fourier shell correlation (FSC) curves showing overall resolution at 2.01 Å at an FSC of 0.143. (**F to H**). Representative resected images of the density maps of the CIC (volume map) including the NTD (residues K42-Q53; **F**), core domain residues Q146-Q164 (**G**), and vDNAs with DTGs (**H**). The maps are shown with the final structural models superimposed.

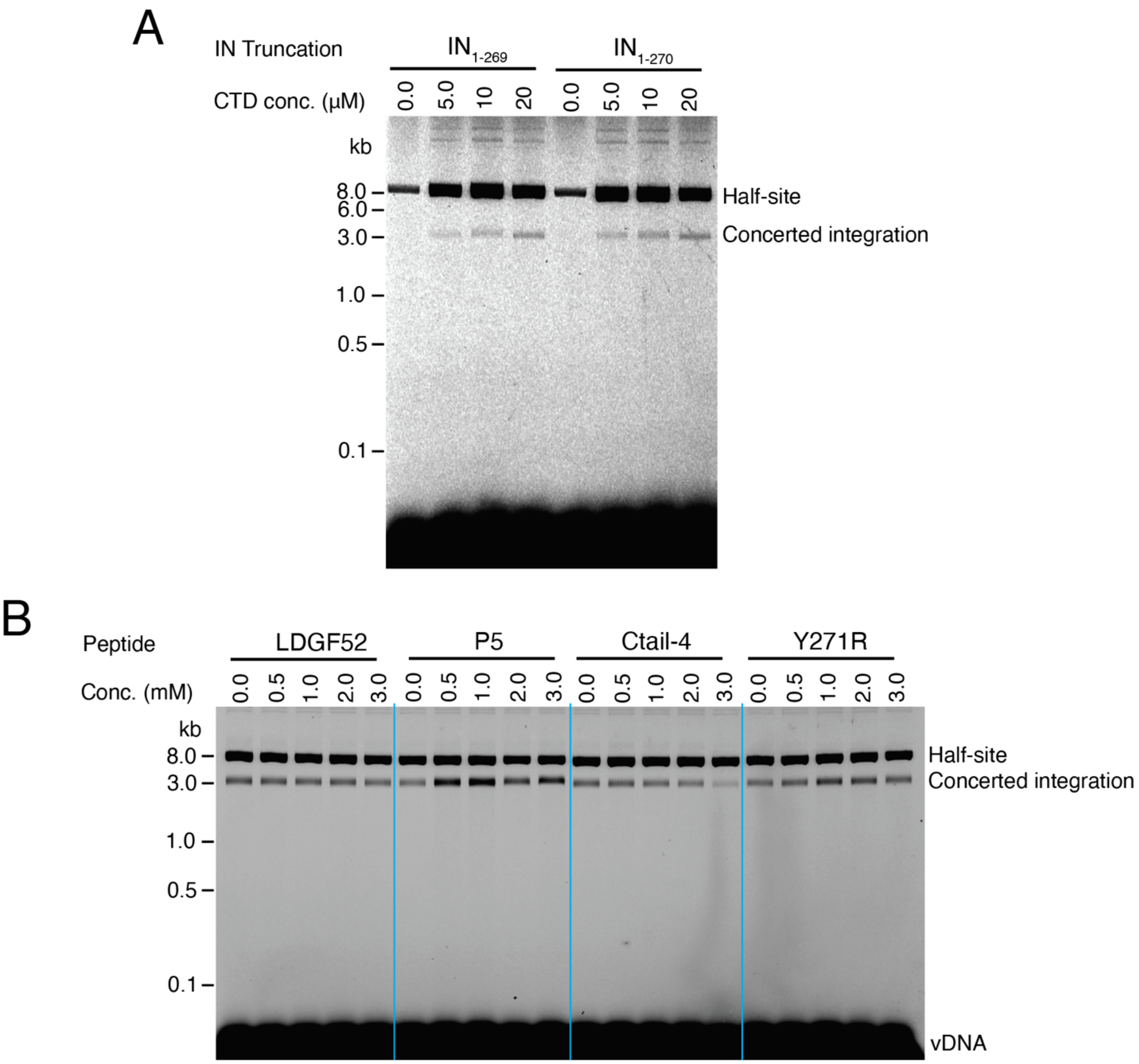

Peptide	Sequence
LDGF52	KPSSEERETEISLKDSTLDN
P5	PKRGRPAATEVKIPKPRGRP
Ctail-4	DYGKQMAGDDCVASRQDED
Y271R	DRGKLMPQDRCVASRQDED

Figures S2. Exogenous CTD moderately stimulates IN C-terminal tail truncation mutant activity. (A) The indicated concentration of CTD was preincubated with either 1.0 μM C-terminal tail truncated Sso7d-IN_{1-269} or Sso7d-IN_{1-270} and 1.0 μM U5-25bp vDNA substrate prior to strand transfer at 37 °C for 2 h. The integration product DNAs were visualized by fluorescence using a Typhoon 8600 scanner. (**B**) The indicated peptides were preincubated with full length Sso7d-IN prior to strand transfer at 37 °C for 2 h. Concentration of peptides are indicated. LDGF52, peptide with random sequence; P5, LEDGF-derived stimulation peptide; Ctail-4, C-terminal tail peptide from 270 to 288; Y271R, similar sequence as Ctail-4 except with 3 amino acids changed. Sequences are shown in the lower panel. The results are representative of three independent experiments.

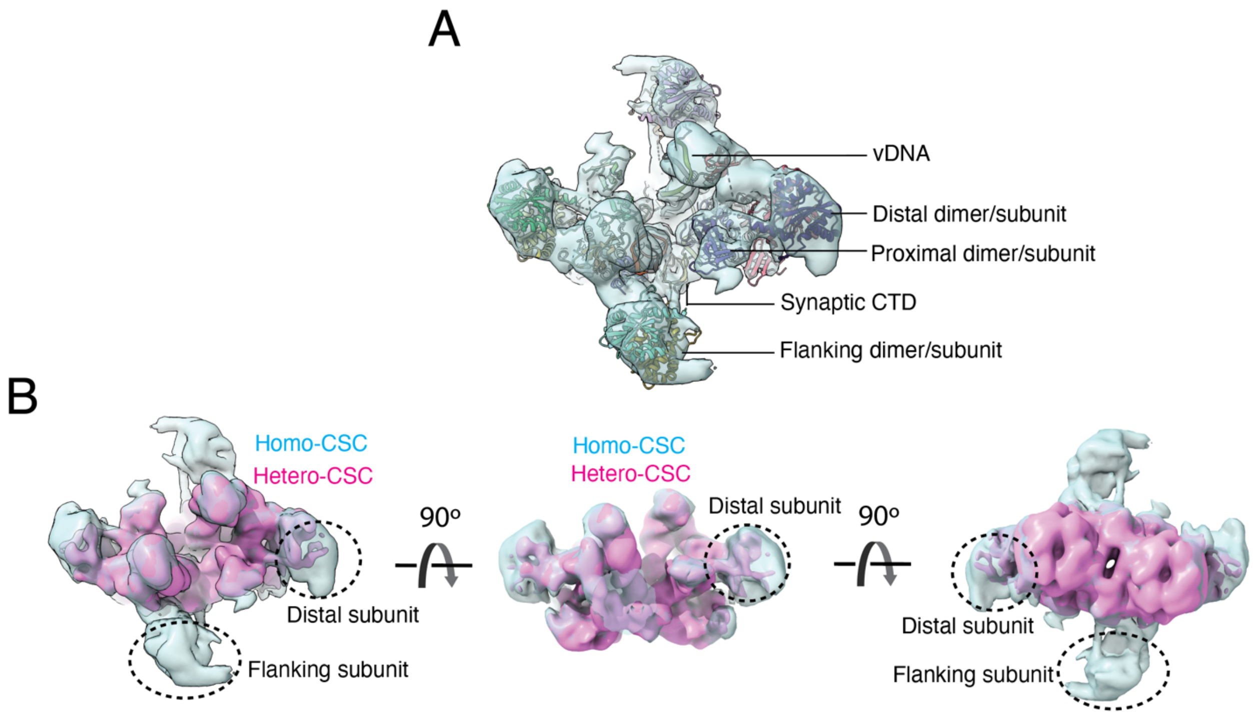

Figure S3. Cryo-EM reconstruction density map comparison. (**A**) Typical density map of the intasome assembled with full-length Sso7d-IN (homo-CSC). The map and intasome structural model are superimposed. (**B**) Density maps of "hetero-intasome" (Hetero-CSC, pink) and "Homo-CSC" (light blue) are superimposed. The missing density at flanking and distal subunit regions in "hetero-CSC" are indicated with circles, indicating those subunits are completely or partially replaced by the exogenous CTD in the assembly.
